# Supplementary material for: Deletion of wfs1 Impairs Oligodendrocyte Precursor Cells Dorsal Distribution and Myelination Through the wfs1-hmgcs1 Axis in Zebrafish
Source: Biology (Basel). 2026 Aug 16;15(16):1402. doi: 10.3390/biology15161402 (PMC13510151; doi:10.3390/biology15161402)
Supplement: Supplementary file 1 [file biology-15-01402-s001.zip › File S1-Transcriptome sequencing data and differentially expressed genes (DEGs) in wfs1-- zebrafish at 5 dpf/1.GO/wfs1KOvsWT/GSEA/WT/SEPTIN_RING/SEPTIN_RING(GO_0005940).html]

Details for gene set SEPTIN\_RING(GO:0005940)[GSEA]


|  || Dataset | deg.sample |
| Phenotype | sample.cls |
| Upregulated in class | WT |
| GeneSet | SEPTIN\_RING(GO:0005940) |
| Enrichment Score (ES) | -0.647948 |
| Normalized Enrichment Score (NES) | -1.844773 |
| Nominal p-value | 0.0 |
| FDR q-value | 0.25706062 |
| FWER p-Value | 0.564 |
Table: GSEA Results Summary

  

Fig 1: Enrichment plot: SEPTIN\_RING(GO:0005940)
  
Profile of the Running ES Score & Positions of GeneSet Members on the Rank Ordered List

  

| PROBE | DESCRIPTION   (from dataset) | GENE SYMBOL | GENE\_TITLE | RANK IN GENE LIST | RANK METRIC SCORE | RUNNING ES | CORE ENRICHMENT || 1 | 556486 | sept4a |  |  | 6511 | 0.536 | -0.1467 | No |
| 2 | 571702 | sept8b |  |  | 9890 | 0.310 | -0.2193 | No |
| 3 | 445325 | sept5b |  |  | 12645 | 0.157 | -0.2888 | No |
| 4 | 323457 | sept2 |  |  | 14860 | 0.053 | -0.3526 | No |
| 5 | 335925 | sept5a |  |  | 15509 | 0.022 | -0.3706 | No |
| 6 | 550129 | sept10 |  |  | 18262 | -0.088 | -0.4475 | No |
| 7 | 321590 | sept8a |  |  | 19001 | -0.121 | -0.4576 | No |
| 8 | 554123 | sept3 |  |  | 19212 | -0.131 | -0.4501 | No |
| 9 | 100000597 | sept7b |  |  | 20105 | -0.171 | -0.4597 | No |
| 10 | 393431 | zgc:63587 |  |  | 20871 | -0.208 | -0.4614 | Yes |
| 11 | 337243 | sept9a |  |  | 21278 | -0.229 | -0.4495 | Yes |
| 12 | 564600 | sept12 |  |  | 22004 | -0.267 | -0.4435 | Yes |
| 13 | 322694 | sept6 |  |  | 23764 | -0.378 | -0.4581 | Yes |
| 14 | 558037 | zgc:162239 |  |  | 25031 | -0.449 | -0.4496 | Yes |
| 15 | 792259 | sept15 |  |  | 31355 | -2.543 | -0.3747 | Yes |
| 16 | 541494 | sept9b |  |  | 31689 | -3.641 | 0.0063 | Yes |
Table: GSEA details
[plain text format]

  

Fig 2: SEPTIN\_RING(GO:0005940)
  
Blue-Pink O' Gram in the Space of the Analyzed GeneSet

  

Fig 3: SEPTIN\_RING(GO:0005940): Random ES distribution
  
Gene set null distribution of ES for
 **SEPTIN\_RING(GO:0005940)**
